# Duplex fluorescence melting curve analysis as a new tool for rapid detection and differentiation of genotype I, II and Bartha-K61 vaccine strains of pseudorabies virus

**DOI:** 10.1186/s12917-018-1697-4

**Published:** 2018-11-28

**Authors:** Zhicheng Liu, Chunhong Zhang, Haiyan Shen, Junying Sun, Jianfeng Zhang

**Affiliations:** Institute of Animal Health, Guangdong Academy of Agricultural Sciences, Key Laboratory of Livestock Disease Prevention of Guangdong Province, Scientific Observation and Experiment Station of Veterinary Drugs and Diagnostic Techniques of Guangdong Province,Ministry of Agriculture, P.R.China, Guangzhou, 510640 Guangdong China

**Keywords:** PRV, Duplex FMCA, Genotyping

## Abstract

**Background:**

Recently, pseudorabies (PR) outbreaks have been reported in a large number of swine herds vaccinated with the Bartha-K61 vaccine in China, the current pseudorabies virus (PRV) belonging to Genotype II is differential genetically from Bartha-K61 vaccine belonging to Genotype I. Furthermore, it has been proved that the Bartha-K61 vaccine cannot provide sufficient protection against the current PRVs in China. Therefore, the accurate and rapid identification of PRVs is essential. The objective of this study is to develop a duplex fluorescence melting curve analysis (FMCA) capable of rapid, simple, high-throughput differentiation of Chinese, European/American and Bartha-K61 vaccine strains of PRV.

**Results:**

Primers 6F/6R and probes P1/P2, combined with three recombinant plasmids p-B (Bartha-K61), p-N (Genotype I), and p-H (Genotype II), were used to establish the Bicolor FMCA. FAM *Tm* values (probe P1) and HEX (probe P2) channels of p-B were used as reference values. *Tm* differences (*ΔTm*) between detected samples and reference plasmid p-B were calculated in each channel. Bartha-K61 vaccine samples had *ΔTm* values of ±1 °C in both FAM and HEX channels, Genotype I samples had *ΔTm* values of ±1 °C in the FAM channel and 4.38 ± 1 °C in the HEX channel, and Genotype II samples had *ΔTm* values of 6.52 ± 1 °C in the FAM channel and 4.38 ± 1 °C in the HEX channel. The minimum detection limit of the duplex FMCA was approximately 1 × 10^0^ copies per reaction for p-B, p-N, and p-H. The duplex FMCA technique was used to detect and different 198 suspected clinical samples, of which 18 (9%) were positive for Genotype II strains and eight (4%) were positive for Bartha-K61 vaccine strains, and the results were compared with sequencing and phylogenetic analyses, which confirmed that the Bicolor FMCA worked correctly for all samples.

**Conclusions:**

In this study, we developed a duplex FMCA of dual-labeled, self-quenched probes that was performed for rapid detection and differentiation of Genotype I, II and Bartha-K61 vaccine strains of PRV. The duplex FMCA was rapid, simple, and high-throughput, and will likely prove useful for molecular epidemiological investigations and pathogen surveillance of PRV.

**Electronic supplementary material:**

The online version of this article (10.1186/s12917-018-1697-4) contains supplementary material, which is available to authorized users.

## Background

Pseudorabies (PR), also known as Aujeszky’s disease (AD), is caused by pseudorabies virus (PRV), which is also known as suid herpesvirus 1 (SuHV-1) or Aujeszky’s disease virus (ADV). PR is an economically important disease in the pig industry worldwide. Swine are the only known natural host of PRV, but nearly all mammals are susceptible to infection [1]. PRV infection is characterised by neurological symptoms and death in newborn piglets, respiratory disorders in fattening pigs, and reproductive failure in pregnant pigs [2]. PR can be controlled using attenuated Bartha-based vaccines and in combination with a worldwide eradication program. However, since the latter part of 2011, PR outbreaks have been reported in a large number of swine herds vaccinated with the Bartha-K61 vaccine in China [3–8]. The Bartha-K61 vaccine did not provide full protection against the current PRVs [3, 9]. Current evidence suggests PRV can be divided into two distinct clusters, with Chinese strains belonging to genotype II and European/American strains in genotype I, which contains Bartha-K61 vaccine [10–13]. Therefore, the accurate differentiation of the PRVs is essential.

Traditionally, etiological diagnostic methods for PR include virus isolation [14], DNA hybridisation [15–17], immunofluorescence [17], loop-mediated isothermal amplification (LAMP) assay [18], real-time polymerase chain reaction (PCR) assay [19], and recombinase polymerase amplification assays (RPA) [20], none of which are able to distinguish between PRV strains. Several assays, including the LAMP assay [21], nanoparticle-assisted PCR assay [22], and two multiplex real-time PCR (qPCR) assays [23], have been developed to differentiate between wild-type (WT) PRV and gene-deleted PRV vaccines. However, these assays are unable to discriminate between WT PRV strains. Triplex real-time PCR [24] is applicable for differential detection of classical, variant and Bartha-K61 vaccine strains of PRV. However, classical PRV strains in the present study include both Chinese strains and European/American PRVs, which are evolutionarily distinct clades [10, 24].

Probe-based fluorescence melting curve analysis (FMCA) is a powerful tool for single-nucleotide polymorphism (SNP) genotyping of target sequences based on melting temperature generated by thermal denaturation of the probe-target hybrid. Recently, FMCA was used in the detection of bacterial resistance [25] and virus genotyping [26]. The aim of the present study was to develop a duplex FMCA capable of rapid, simple, high-throughput differentiation of Chinese, European/American and Bartha-K61 vaccine strains of PRV.

## Results

**Fig. 1:**
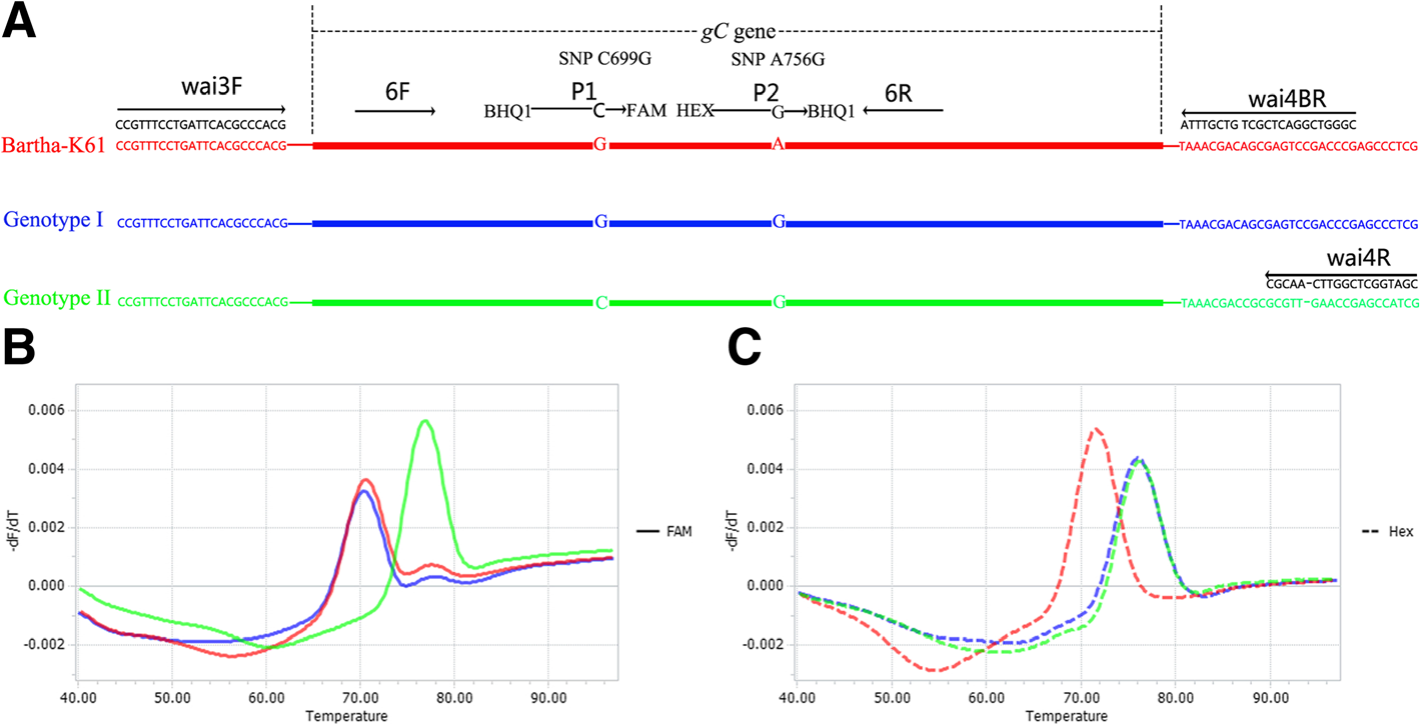
Schematic illustration of the duplex FMCA method. (**a**) Relative binding positions of primers and probes along the *gC* gene of PRV. Melting peak calculation by derivative plotting -dF/dT versus temperature in the FAM channel (**b**) and the HEX channel (**c**). Red, blue, and green lines represent Bartha-K61 vaccine, European/American (Genotype I), and Chinese (Genotype II) strains, respectively

The developed Bicolor FMCA of dual-labeled, self-quenched probes was able to discriminate 92 samples within 2 h when the template DNA was ready. This assay could identify Chinese strains (Genotype II), European/American strains (Genotype I) and Bartha-K61 vaccine strains of PRV based on *Tm* value and fluorescence type, as shown in Fig. 1b and Fig. 1c. For each sample, two *Tm* values were obtained from the FAM and HEX channels, corresponding to probes P1 and P2, respectively. P1 was designed to match Genotype II but mismatch Genotype I (including Bartha-K61 vaccine strains), while P2 was designed to match Genotype I and Genotype II but mismatch Bartha-K61 vaccine strains. As shown in Table 1, in the FAM channel, Genotype I (including Bartha-K61 vaccine strains) yielded *Tm* values of 70.41 ± 0.49 °C (0.49 °C = 3SD, *n* = 63), and Genotype II *Tm* values were 76.93 ± 0.48 °C (*n* = 32). In the HEX channel, Bartha-K61 vaccine strains gave *Tm* values of 71.62 ± 0.18 °C (n = 32), while Genotype I and Genotype II *Tm* values were 76.00 ± 0.48 °C (*n* = 63). In each assay, recombinant plasmids p-B (Bartha-K61 Vaccine), p-N (Genotype I), and p-H (Genotype II) were used as positive controls, and the NTC sample served as the negative control. In order to simplify data analysis, *Tm* values in FAM and HEX channels of recombinant plasmid p-B was used as reference values, and *Tm* differences (*ΔTm*) between detected samples and reference plasmid p-B were calculated in each channel. As shown in Table 1, *Tm* values were highly reproducible, with a maximum 3SD value of 0.49 (Δ*Tm* = 6.52 °C, FAM channel) and 0.48 (Δ*Tm* = 4.38 °C, HEX channel). Bartha-K61 vaccine samples had *ΔTm* values of ±1 °C in both the FAM and HEX channels, Genotype I samples had *ΔTm* values of ±1 °C in the FAM channel and 4.38 ± 1 °C in the HEX channel, and Genotype II samples had *ΔTm* values of 6.52 ± 1 °C in the FAM channel and 4.38 ± 1 °C in the HEX channel.

**Table 1:** Reproducibility testing of Bicolor FMCA for each positive control sample

Name	No. of times tested	*Tm* value (°C, Mean ± 3SD)	
		P1 (FAM channel)	P2 (HEX channel)
Bartha-K61	32	70.41 ± 0.49 ^a,^*	71.62 ± 0.18*
NIA3	31		76.00 ± 0.48^b^
HDDJ	32	76.93 ± 0.48	

^a^*Tm* value calculated based on the number of Bartha-K61 strains and NIA3 strains tested in the FAM channel. ^b^*Tm* value calculated based on the number of NIA3 strains and HDDJ strains tested in the HEX channel. *: The asterisk symbol was indicated significant difference between the melting temperature of two genotypes in same channel (*P*<0.001).

### Sensitivity, specificity and reproducibility

Serial dilutions of the three recombinant plasmids p-B, p-N, and p-H ranged from 1 × 10^9^ to 1 × 10^0^ copies/μL when tested with the Bicolor FMCA, and fluorescence signals corresponding to melting curves were obtained between 1 × 10^0^ and 1 × 10^9^ copies per reaction for each plasmid. No specific signals were detected and corresponding melting curves were not obtained with the negative control (Additional file 1). Therefore, the detection limit of the Bicolor FMCA was approximately 1 × 10^0^ copies per reaction for recombinant plasmids p-B, p-N, and p-H.

**Fig. 2:**
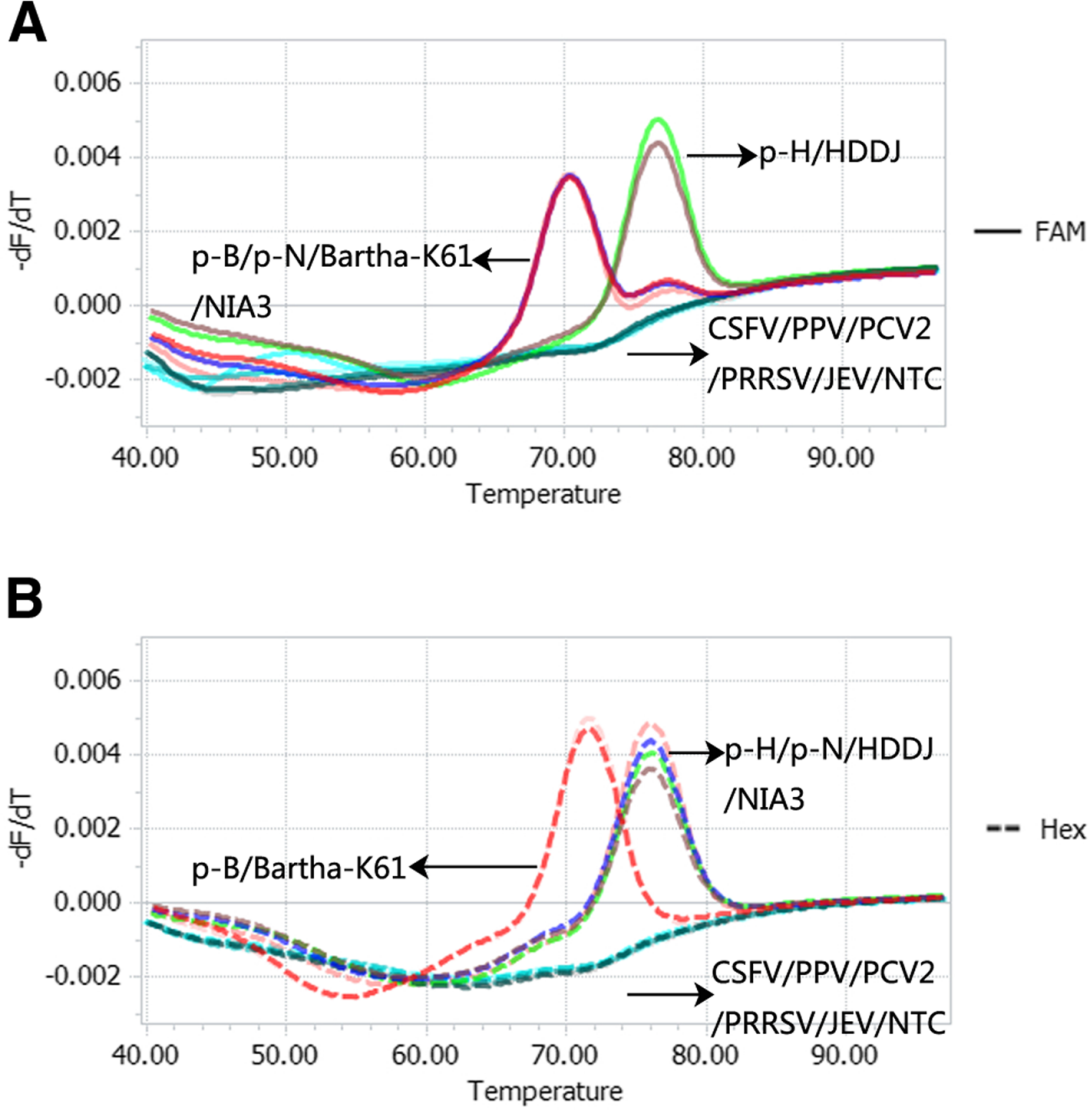
Specificity of the duplex FMCA method. Melting curves obtained from duplex FMCA in the FAM channel (**a**) and the HEX channel (**b**) with 12 samples comprising 3 PRV positive samples, and five other viruses known to cause similar clinical symptoms in pig. Reference recombinant plasmids p-B (Bartha-K61 vaccine), p-N (Genotype I), and p-H (Genotype II) served as positive controls corresponding to red, blue, and green lines, and a No Template Control (NTC) served as the negative control (grey line)

The specificity of the Bicolor FMCA was validated using DNA or cDNA samples from three PRV isolates, five other viruses known to cause similar clinical symptoms in pig, and three positive control plasmids (Fig. 2). Based on the data analysis method described above, detected samples included two Bartha-K61 vaccine samples (including plasmid p-B and Bartha-K61), two Genotype I samples (including plasmid p-N and NIA3), and two Genotype II samples (including plasmid p-H and HDDJ). However, no specific melting peak was detected with other non-targeted pig viruses such as CSFV, PPV, PCV2, PRRSV, and JEV in the FAM and HEX channels. These results suggest that the designed primers and probes were highly specific and selective for their target viruses and exhibited no cross-reactivity with other viruses.

To evaluate the run-to-run reproducibility of the Bicolor FMCA, given the narrow *Tm* windows, the assay was run on different days using PRV Bartha-K61, NIA3, and HDDJ samples. Melting curve analysis for samples run on different days yielded a maximum 3SD value for *Tm* of no more than 0.5 for all melting peaks (Table 1), demonstrating the high reproducibility of this assay.

### Agreement among the bicolor FMCA, sequencing and phylogenetic analysis

Of 198 suspected clinical samples were tested by the Bicolor FMCA technique, 18 (9%) were positive for Genotype II strains and eight (4%) were positive for Bartha-K61 vaccine strains (Table 2), but Genotype I strains were not detected.

**Table 2:** *Tm* values from Bicolor FMCA of the PRV strains used in this study

Name	GenBank accession no.	*Tm* value (°C)		*ΔTm* value (°C)^a^		Genotype	Interpretation
		P1 (FAM channel)	P2 (HEX channel)	P1 (FAM channel)	P2 (HEX channel)		
p-B	—	70.53	71.61	0	0	Genotype I (Bartha-K61)	reference control/plasmid
p-N	—	70.53	75.99	0	4.38	Genotype I	positive control/plasmid
p-H	—	76.88	75.98	6.35	4.37	Genotype II	positive control/plasmid
Bartha-K61	JF797217.1	70.50	71.59	-0.03	-0.02	Genotype I (Bartha-K61)	positive sample
NIA3	KU900059.1	70.28	75.96	-0.25	4.35	Genotype I	positive sample
HDDJ	MF434048	76.70	76.00	6.17	4.39	Genotype II	positive sample/field sample
GDGZ	MF434045	76.75	75.84	6.22	4.23	Genotype II	field sample
GDZQ	MF434042	76.93	76.20	6.4	4.59	Genotype II	field sample
GDHY	MF434044	77.13	76.21	6.6	4.6	Genotype II	field sample
GDHY2	MF434038	76.70	76.00	6.17	4.39	Genotype II	field sample
GDZQ2	MF434039	76.95	76.04	6.42	4.43	Genotype II	field sample
GDSD	MF434043	76.74	76.04	6.21	4.43	Genotype II	field sample
JXGZ	MF434047	76.67	75.97	6.14	4.36	Genotype II	field sample
JXGZ2	MF434036	76.63	75.93	6.1	4.32	Genotype II	field sample
LC	MF434047	76.69	75.98	6.16	4.37	Genotype II	field sample
HNCS	MF434046	76.51	75.81	5.98	4.20	Genotype II	field sample
GDHY8	MH053134	77.27	76.16	6.74	4.55	Genotype II	field sample
GDGZ2	MH053135	76.87	76.15	6.34	4.54	Genotype II	field sample
GDBL	MH053136	76.75	76.03	6.22	4.42	Genotype II	field sample
SXDT	MH053137	76.14	76.24	5.61	4.63	Genotype II	field sample
HNCS4	MH053138	76.99	76.08	6.46	4.47	Genotype II	field sample
GDHZ	MH053139	76.91	76.00	6.38	4.39	Genotype II	field sample
GDQY	MH053140	76.92	76.01	6.39	4.40	Genotype II	field sample
GDHY5	—^b^	70.49	71.60	-0.04	-0.01	Genotype I (Bartha-K61)	field sample
GDZQ4	—^b^	70.51	71.61	-0.02	0	Genotype I (Bartha-K61)	field sample
JXGZ4	—^b^	70.57	71.66	0.03	0.05	Genotype I (Bartha-K61)	field sample
GDSG	—^b^	70.56	71.61	0.03	0	Genotype I (Bartha-K61)	field sample
GDMM	—^b^	70.45	71.56	-0.08	-0.05	Genotype I (Bartha-K61)	field sample
GDFS	—^b^	70.71	71.61	0.18	0	Genotype I (Bartha-K61)	field sample
GDZS	—^b^	70.48	71.57	-0.05	-0.04	Genotype I (Bartha-K61)	field sample
GDQY3	—^b^	70.55	71.64	0.02	0.03	Genotype I (Bartha-K61)	field sample

^a^*ΔTm* = *Tm* value of detected samples minus *Tm* of reference plasmid p-B in each channel. ^b^The detected strains shared 100% identities in *gC* nucleotide sequence to Bartha-K61 strain (JF797217.1).

To confirm that the clustering pattern resulting from the difference-melt curves of the different genotypes, the *gC* gene of each sample was sequenced and compared with consensus sequences (Additional file 2). Ten control sequences were included from the GenBank databases when sequences were aligned. Sequences of the 26 positive samples aligned well with the GenBank controls and with each other, confirming that the Bicolor FMCA worked correctly for all samples (Additional files 2 and 3). The relationship between individuals is shown in the cladogram.

To additionally confirm the eight Bartha-K61 strains, the fragment encompassing the *gE* gene was amplified by the primers gE-F and gE-R. The result showed that no fragment was amplified from the eight Bartha-K61 strains (Additional file 4). Furthermore, the eight samples were obtained from Bartha-K61–vaccinated pigs.

## Discussion

Recently, PR outbreaks have been reported in a large number of swine herds vaccinated with the Bartha-K61 vaccine in China [3–8]. And the phylogenetic analysis based on full-length PRV sequences and based on only the *gC* gene and other genes revealed that PRV could be divided into two major genotypes, with Genotype I containing isolates from Europe, America, Australia, and Asia, and Genotype II consisting mainly of isolates from China, including classical strains such as Fa and Ea, as well as recently emerged variant strains [10–13]. Furthermore, it has been proved that the Bartha-K61 vaccine belonging to Genotype I cannot provide full protection against the current PRVs in China belonging to Genotype II [3, 9]. Therefore, it is very important to develop a method to identify pigs infected with PRV genotypes or immunized with the PRV Bartha-K61 vaccine strain.

In this study, we developed a simple and rapid method for detection and differentiation of PRV strains using a duplex FMCA approach with dual-labeled, self-quenched probes. This method proved to be easier, faster and more straightforward than conventional differentiation methods such as virus isolation combined with sequence analysis, which is often used to distinguish different strains [10].

The TaqMan probe has a random coil conformation that leads to fluorescence quenching or only weak fluorescence unless the probe is either hybridised with its target or digested. In classic TaqMan real-time PCR, generation of the fluorescence signal is based on probe degradation by the 5′-3′ exonuclease activity of DNA Taq polymerase in each round of amplification [27]. In FMCA, after PCR, a larger amount of fluorescence is emitted upon probe hybridisation with target sequences because the fluorophore is no longer in close proximity to the quencher. In this study, *TaKaRa LA Taq* DNA Polymerase combined *Taq* DNA Polymerase with 5′-3′ exonuclease activity and a DNA proofreading polymerase with 3′-5′ exonuclease activity were employed during PCR analyses. Hence, as described previously [28], the effects of hydrolysis of the probe during PCR on the melting curve profile require assessment. We compared the melting curves and melting peaks obtained from experiments in which probes were added either before or after the PCR run (Additional file 5). The results showed that the background fluorescence intensities in FAM and HEX channels increased when probes were added before the PCR run at the beginning of the melting curve analysis (Additional file 5.A and C) because some probes were digested during PCR by the 5′-3′ exonuclease activity of *TaKaRa LA Taq* DNA Polymerase. However, this did not affect the *Tm* value of the melting curve or the melting peak of FAM and HEX channels (Additional file 5). Additionally, this did not affect the −dF/dT value of the FAM channel when temperature equal to the *Tm* value (Additional file 5B), or slightly below the −dF/dT value of the HEX channel (Additional file 5.D). In summary, *TaKaRa LA Taq* was shown to be suitable for this assay.

In this study, the limit of detection of the Bicolor FMCA was determined to be 1 × 10^0^ copies per reaction for recombinant plasmids p-B, p-N and p-H. Some factors contributed greatly to this. Firstly, only one pair of primers was used during the PCR process, and post-multiplex FMCA could reduce some of the nonspecific interactions if two or more pairs of primers are used [29]. Secondly, the asymmetric PCR post-multiplex FMCA was optimised in terms of the ratio of primer 6F to 6R. In order to maximise the amplified single strand used to hybridise with the probes while retaining the basic PCR reaction conditions, the ratio of primers is important, as described previously [26, 28, 30–38]. The results showed that detection by the Bicolor FMCA was highest when the ratio of primer 6F to 6R was 1:10 (0.04 μM: 0.4 μM) and probe P1 and P2 was 0.2 μM. Thirdly, the PCR amplification system containing *TaKaRa LA Taq* and GC Buffer I improved the PCR amplification efficiency significantly over the conventional PCR amplification system for the high GC content of the PRV genome.

**Fig. 3:**
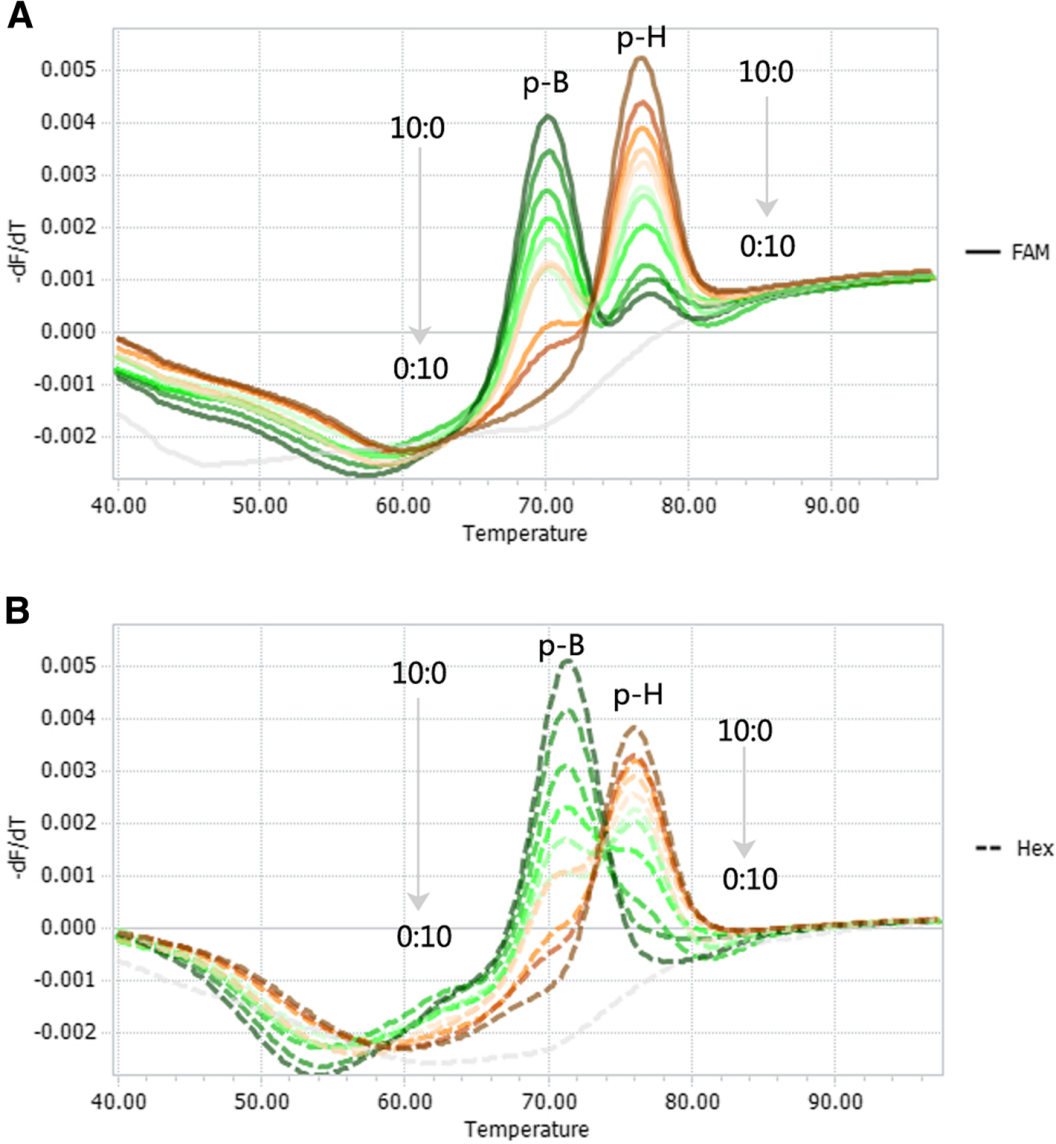
Simultaneous detection of PRV Bartha-K61vaccine and Genotype II strains in mixed infections. Two co-existing genotypes were detected in the FAM channel (**a**) and the HEX channel (**b**). Melting curves of artificial plasmid templates containing p-B and p-H at various ratios (10:0, 9:1, 8:2, 7:3, 6:4, 5:5, 4:6, 3:7, 2:8, 1:9, 0:10) were tested. The overall template concentration was 10^8^ copies per reaction. The NTC negative control is represented by a grey line

Since the 1990s, most pig herds in China have been immunised with the Bartha-K61 vaccine, which is a live attenuated strain (Bartha, 1961) [3]. However, co-infection with more than one PRV genotype has not been widely reported, possibly due to limited differential detection methods in the past. Using the dual presence of PRV recombinant plasmids p-B and p-H as an example, we tested the ability to distinguish the two genotypes when they are simultaneously present in the same reaction. By varying their relative ratios, the results showed that levels as low as 10% (1:9) of one genotype in the presence of 90% (9:1) of the other genotype could be detected when the overall copy number was 10^8^ copies per reaction (Fig. 3).

It should be pointed out that this method is only applicable for genotypes from Chinese, European/American and Bartha-K61 vaccine strains of PRV because it was developed based on SNP differences. Thus, the method is not suitable for the genotyping of PRV genetically engineered vaccine strains such as HB-98 (MF434040), HB-2000 (MF434041), and SA215 (EU719635.1) strains. Also, the method is unable to subdivide either Chinese strain into ancient and recently emerged strains, or European/American PRVs into six subgroups [10]. A more inclusive method needs to be developed in the future for this purpose.

## Conclusions

In summary, the newly developed Duplex FMCA is a specific and sensitive method for the differential detection of Chinese, European/American and Bartha-K61 vaccine strains of PRV, and will likely prove useful for molecular epidemiological investigations and pathogen surveillance of PR.

## Methods

### Strain collection and DNA extraction

A total of 28 PRV samples (Table 2) and five other viruses were tested in this study. Three positive strains were included to establish the Bicolor FMCA: PRV Bartha-K61 (Bartha-K61 Vaccine, Boehringer CO. LTD, Germany), NIA3 (Genotype I, the NIA3 strain), and HDDJ (Genotype II, the HDDJ strain) and other 25 field samples were isolated in our laboratory; the other five viruses are known to cause similar clinical symptoms in pigs and consisted of classical swine fever virus (CSFV) C strain (Winsun Bio, Guangzhou, China), porcine parvovirus (PPV) GD3 strain (isolated in our laboratory), porcine circovirus type 2 (PCV2) BL strain (isolated in our laboratory), porcine reproductive and porcine respiratory syndrome virus (PRRSV) GDr180 strain (Winsun Bio, Guangzhou, China), and Japanese encephalitis virus (JEV) SA14–14-2 strain (Wuhan Keqian Biology., Ltd., Wuhan, China). Viral RNA/DNA was extracted using the MiniBEST Viral RNA/DNA Extraction Kit version 4.0 (Takara, Dalian, China) according to the manufacturer’s instructions.

### Primer and probe design

Main primers and probes were designed to target the *gC* gene of PRV (Fig. 1a). Primers 6F (5’-GCCAACGCCTCCCTCGCCCAC-3′) and 6R (5’-CGACGCACACCGCCCGGAAGG-3′), and probes P1 (BHQ1–5’-CGCCAACGGCACCGAGGTCCG**C**AGCGC-3’-FAM) and P2 (HEX-5’-TCGGCCTGAGCGCGCC**G**CCCGTC-3’-BHQ1; differential nucleotides are underlined) were used to establish the Bicolor FMCA. Probe P1 was designed to differentiate between Genotype II and Genotype I strains (including Bartha-K61 vaccine strains), and probe P2 was designed to distinguish Bartha-K61 vaccine strains from other strains. The single nucleotide polymorphisms (SNPs, Fig. 1a), C699G (probe P1) and A756G (probe P2), in the *gC* gene, were chose based on a sequence alignment of all available PRV *gC* gene by a BLAST search in GenBank databases from the National Center for Biotechnology Information (NCBI). The primers wai3F (5’-CCGTTTCCTGATTCACGCCCACG-3′) and wai4R (5’-CGATGGCTCGGTTCAACGC-3′) and wai4BR (5’-CGGGTCGGACTCGCTGTCGTTTA-3′) were used to generate recombinant plasmids, which were used to determine the limit of detection (LOD) of the assay. The primers were also used for sequencing of the g*C* gene. Two reverse primers wai4R and wai4BR bind to similar regions of the PRV genome, but primer wai4R is specific for Chinese strains (Genotype II), combined with primer wai3F, used to amplify a 1594-bp amplicon; while primer wai4BR is specific for European/American strains (Genotype I), combined with primer wai3F, used to amplify a 1577-bp amplicon. This experimental design could allow both PRV genotypes (I and II) to be amplified.

The primers gE-F (5’-TTGAGACCATGCGGCCCTTTCTGCT-3′) and gE-R (5′- GACCGGTTCTCCCGGTATTTAAGCG-3′) were used to confirm the Bartha-K61 strains detected in field samples. The primers amplified no fragment from Bartha-K61 strain, or a 1763-bp fragment encompassing the *gE* gene of PRV wild strains, respectively.

All primer and probe sequences were analysed using BLAST to confirm specificity.

### Duplex real-time PCR and melting curve analysis

For each sample, 1 μL of template DNA was added to a 10 μL PCR mixture containing 1 × GC Buffer I, 400 μM of each deoxynucleoside triphosphate (dNTP), 0.04/0.4 μM primers 6F/6R, 0.2 μM probe P1/P2, and 0.5 U of TaKaRa LA Taq (Takara, Dalian, China). A No Template Control (NTC) was used as the negative control. Amplification and melting curve analysis was performed in a Roche LightCycler 96 System (Roche, Switzerland) with initial denaturation at 95 °C for 3 min, followed by amplification using 55 cycles of 95 °C for 20 s, 60 °C for 20 s, and 72 °C for 20 s. Melting curve analysis was initiated with denaturation at 95 °C for 10 s, followed by hybridisation at 40 °C for 60 s and a stepwise temperature increment from 40 °C to 97 °C at an Ramp rate of 0.13 °C/s with a duration of 1 s and five readings per °C. Fluorescence was measured at each step in both FAM and HEX channels. Melting curves were obtained by plotting the negative derivative of the fluorescence intensity with respect to temperature (−dF/dT) versus temperature (T). The *Tm* value from each probe was automatically obtained by identifying the peak of the corresponding melting curve. For best results, 11 ratios of forward primers to reverse primers (0.5:10, 1:10, 2:10, 3:10, 4:10, 5:10, 6:10, 7:10, 8:10, 9:10, 10:10) and four concentrations of two probes (0.1, 0.2, 0.3, 0.4 μM) were set. The optimization of *Tm* (s) was at a best ratio of 0.04/0.4 μM primers 6F/6R and a concentration of 0.2 μM probe P1/P2.

### Analytical sensitivity

The analytical sensitivity of the Bicolor FMCA was estimated by serial dilution experiments using three recombinant plasmids p-B, p-N, and p-H. The three plasmids were generated using the pMD18-T Vector Cloning Kit (Takara, Dalian, China), and the insert fragments of the plasmids were amplified from Bartha-K61, NIA3, and HDDJ using primers wai3F/wai4R/wai4BR with the TaKaRa LA PCR Kit version 2.1, respectively. Each 25 μL PCR contained 1 × GC Buffer I, 400 μM of each deoxynucleoside triphosphate (dNTP), 0.4 μM primers wai3F/wai4R/wai4BR, 1.25 U of TaKaRa LA Taq (Takara, Dalian, China), and 2 μL of template DNA. Thermal conditions involved initial denaturation at 95 °C for 3 min, followed by amplification using 45 cycles of 95 °C for 40 s, 62 °C for 40 s, and 72 °C for 90 s. Each plasmid was 10-fold serially diluted with water to concentrations of 1 × 10^9^ copies/μL to 1 × 10^0^ copies/μL. The limit of detection for each genotype was determined from the lowest concentration measured by the Bicolor FMCA.

### Specificity and reproducibility

In the Bicolor FMCA, primers 6F/6R and probes P1/P2 were tested for specificity using three PRV positive samples including Bartha-K61, NIA3, HDDJ, and five other viruses known to cause similar clinical symptoms in pig including CSFV C strain, PPV GD3 strain, PCV2 BL strain, PRRSV GDr180 strain, JEV SA14–14-2 strain. DNA samples from three recombinant plasmids p-B, p-N, and p-H were used as positive controls. To evaluate the reproducibility of the assay, Bicolor FMCA was performed on different days using different DNA templates of each positive control sample PRV Bartha-K61, NIA3, and HDDJ. The *Tm* value of each genotype was measured in each channel.

### Screening and genotyping of clinical samples

The Bicolor FMCA was used to screen 198 suspected clinical samples received for laboratory diagnosis of PRV infection from different geographical locations in China. For confirmation of Bicolor FMCA results, each sample was rechecked by virus isolation, followed by DNA sequencing for positive samples.

The genotype of each sample was determined by sequencing using primers wai3F/wai4R/wai4BR with the PCR protocol described as above. PCR products were subjected to bi-directional sequencing (Sangon, Shanghai, China) and the results were analysed using Clustal W with BioEdit software (version 7.0.0, Tom Hall; Ibis Biosciences, Carlsbad, CA, USA). Phylogenetic analysis was performed by the neighbour-joining method with 1000 bootstrap replicates, based on the Kimura 2-parameter model, with joining comparison of the positive samples and control *gC* gene sequences using MEGA 5.2 software [39].

The Bartha-K61 strains detected in field samples was additionally confirmed based on the deleted *gE* gene by the primers gE-F and gE-R. The PCR protocol was following the amplified *gC* gene described above.

## Additional files

Additional file 1: Sensitivity of the duplex FMCA method. Melting curves from duplex FMCA with recombinant control plasmids (A) p-B, (C) p-N, and (E) p-H in the FAM channel, and (B) p-B, (D) p-N, and (F) p-H in the HEX channel, ranging from 1 × 10^0^ to 1 × 10^9^ copies per reaction (from bottom to top). (TIF 1335 kb)

Additional file 2: Alignment of *gC* genes from the detected PRV samples used for Bicolor FMCA. Strains indicated with black discs were used for Bicolor FMCA. (TIF 1008 kb)

Additional file 3: Phylogenetic analysis (B) of *gC* genes from the detected PRV samples used for Bicolor FMCA. Strains indicated with black discs were used for Bicolor FMCA. (TIF 203 kb)

Additional file 4: The Bartha-K61 strains detected in field samples were identified by PCR. The PCR results showed no fragments were amplified from the eight Bartha-K61 strains detected in field samples using the primers gE-F and gE-R. Lane 1, DL 2000 marker; Lane 2, Bartha-K61; Lane 3, NIA3; Lane 4, HDDJ; Lane 5, NTC; Lane 6, GDHY5; Lane 7, GDZQ4; Lane 8, JXGZ4; Lane 9, GDSG; Lane 10, GDMM; Lane 11 GDFS; Lane 12, GDZS; Lane 13, GDQY3. (TIF 366 kb)

Additional file 5: Influence of probe hydrolysis on the melting curve profile. (A) Melting curves and (B) Melting peaks in the FAM channel, and (C) Melting curves and (D) Melting peaks in the HEX channel for recombinant plasmid p-H. Red and green lines indicate the addition of probes P1 and P2 before and after PCR for plasmid p-H, and blue and pink lines indicate the addition of probes P1 and P2 before and after PCR for NTC. (TIF 1001 kb)

## Abbreviations

FMCA: Fluorescence melting curve analysis
PR: Pseudorabies
PRV: Pseudorabies virus
SNP: Single-nucleotide polymorphism
