# Supplementary figures and images for: Duplex fluorescence melting curve analysis as a new tool for rapid detection and differentiation of genotype I, II and Bartha-K61 vaccine strains of pseudorabies virus
Source: BMC Vet Res. 2018 Nov 28;14:372. doi: 10.1186/s12917-018-1697-4 (PMC6264625; doi:10.1186/s12917-018-1697-4)

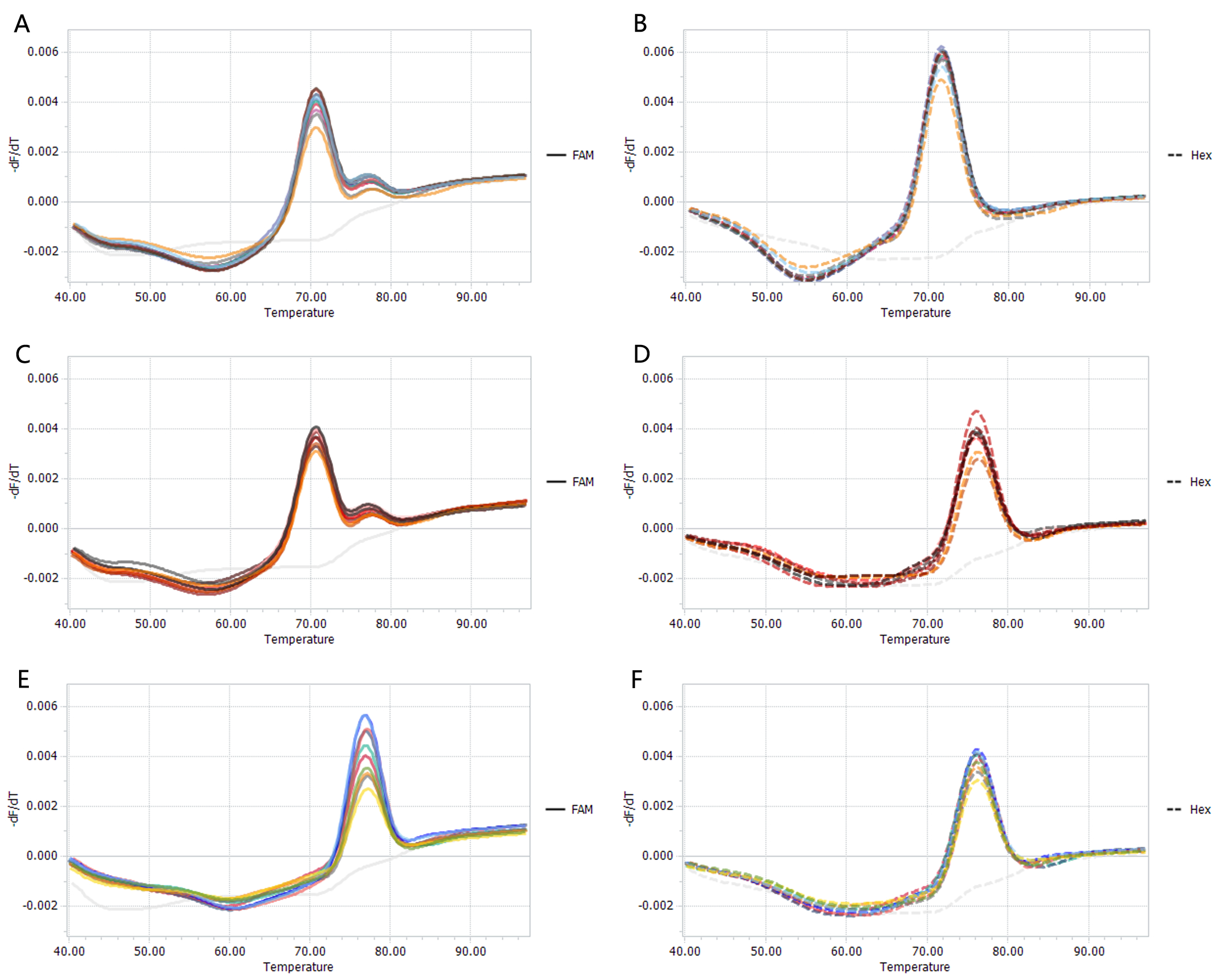

Supplement: Supplementary file 1 — Sensitivity of the duplex FMCA method. Melting curves from duplex FMCA with recombinant control plasmids (A) p-B, (C) p-N, and (E) p-H in the FAM channel, and (B) p-B, (D) p-N, and (F) p-H in the HEX channel, ranging from 1 × 100 to 1 × 10^9^ copies per reaction (from bottom to top). (TIF 1335 kb) [file 12917_2018_1697_MOESM1_ESM.tif]

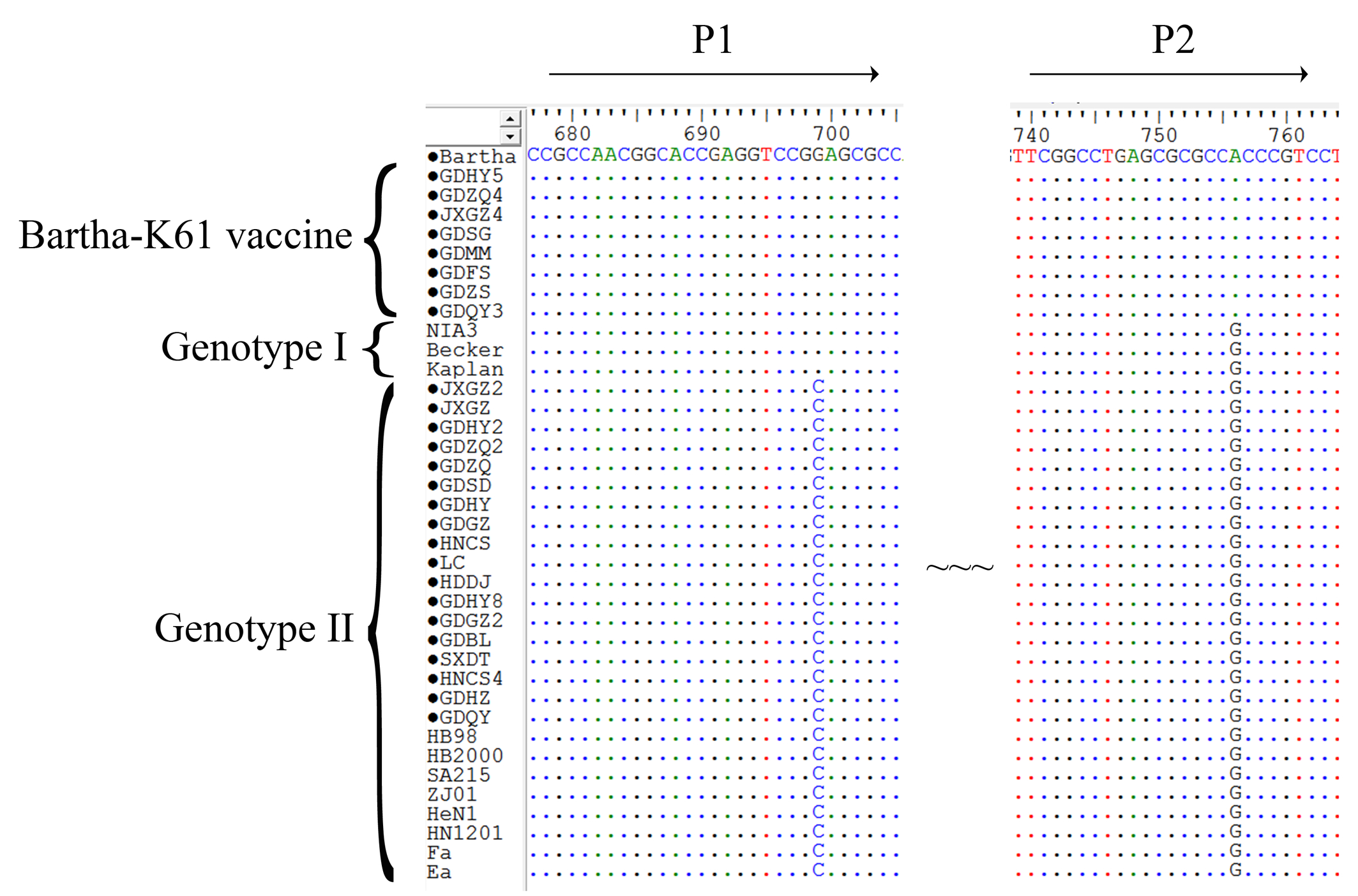

Supplement: Supplementary file 2 — Alignment of gC genes from the detected PRV samples used for Bicolor FMCA. Strains indicated with black discs were used for Bicolor FMCA. (TIF 1008 kb) [file 12917_2018_1697_MOESM2_ESM.tif]

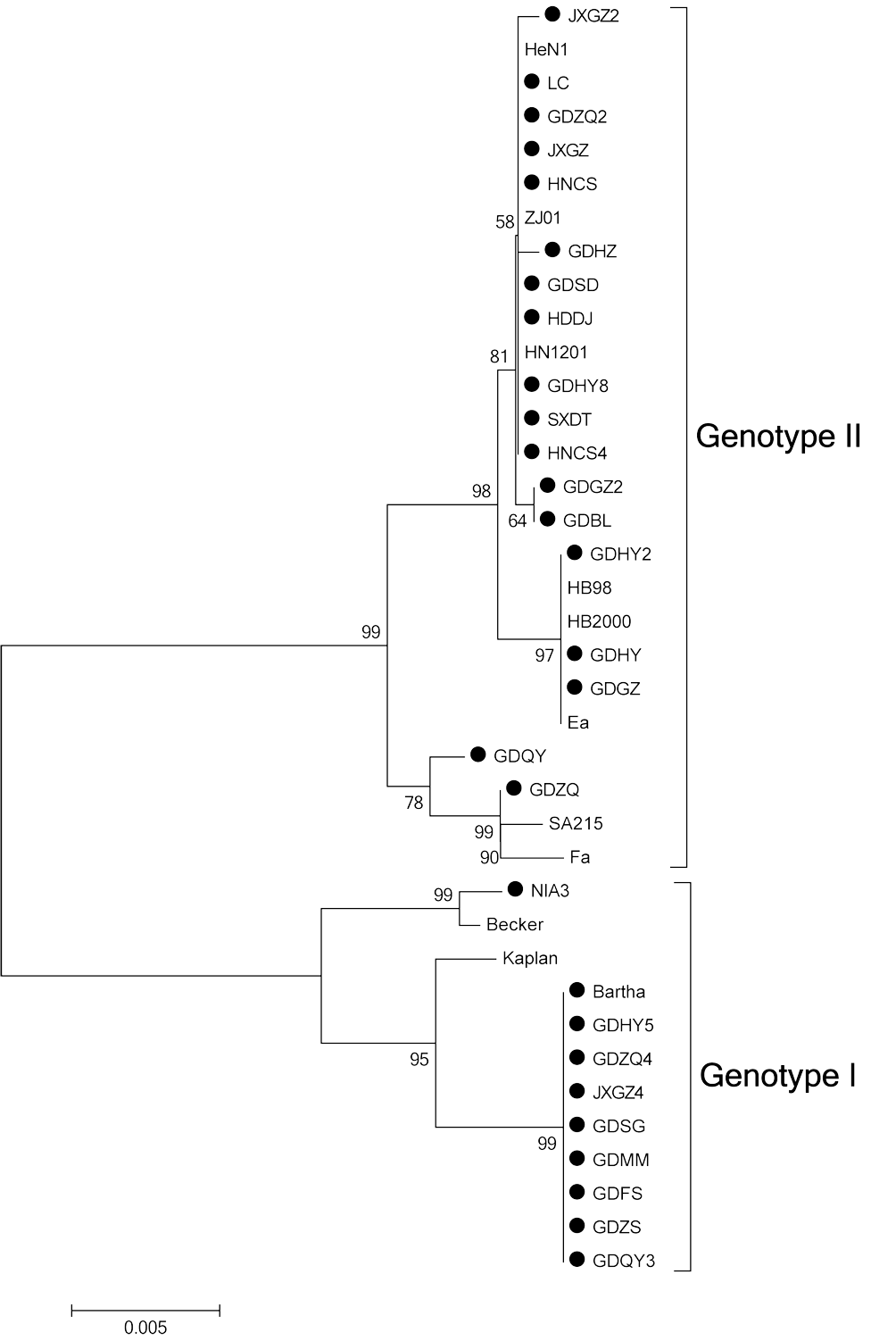

Supplement: Supplementary file 3 — Phylogenetic analysis (B) of gC genes from the detected PRV samples used for Bicolor FMCA. Strains indicated with black discs were used for Bicolor FMCA. (TIF 203 kb) [file 12917_2018_1697_MOESM3_ESM.tif]

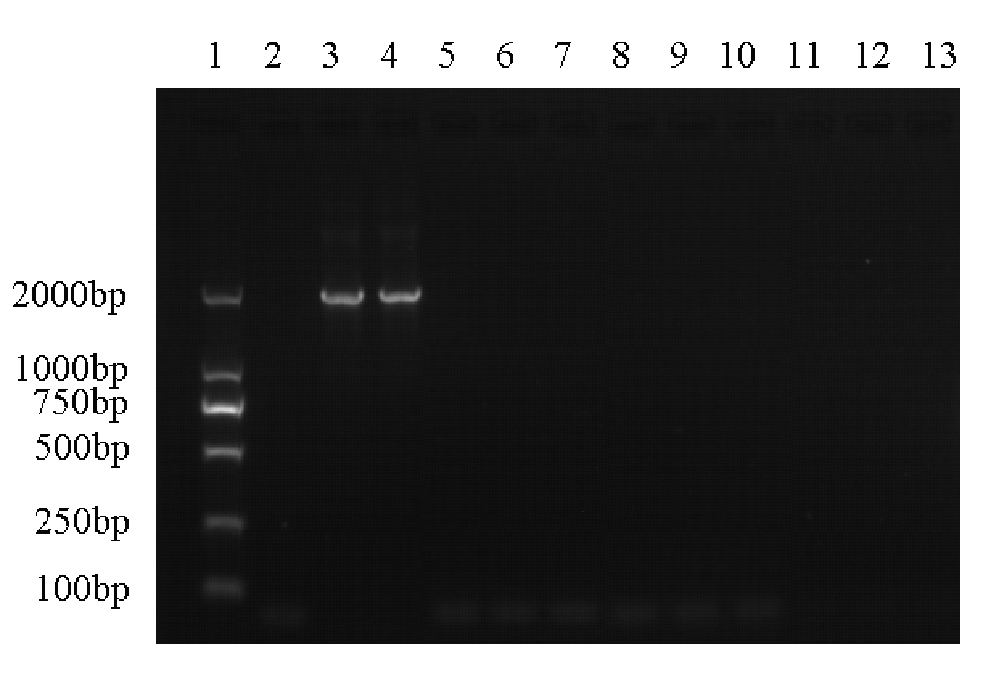

Supplement: Supplementary file 4 — The Bartha-K61 strains detected in field samples were identified by PCR. The PCR results showed no fragments were amplified from the eight Bartha-K61 strains detected in field samples using the primers gE-F and gE-R. Lane 1, DL 2000 marker; Lane 2, Bartha-K61; Lane 3, NIA3; Lane 4, HDDJ; Lane 5, NTC; Lane 6, GDHY5; Lane 7, GDZQ4; Lane 8, JXGZ4; Lane 9, GDSG; Lane 10, GDMM; Lane 11 GDFS; Lane 12, GDZS; Lane 13, GDQY3. (TIF 366 kb) [file 12917_2018_1697_MOESM4_ESM.tif]

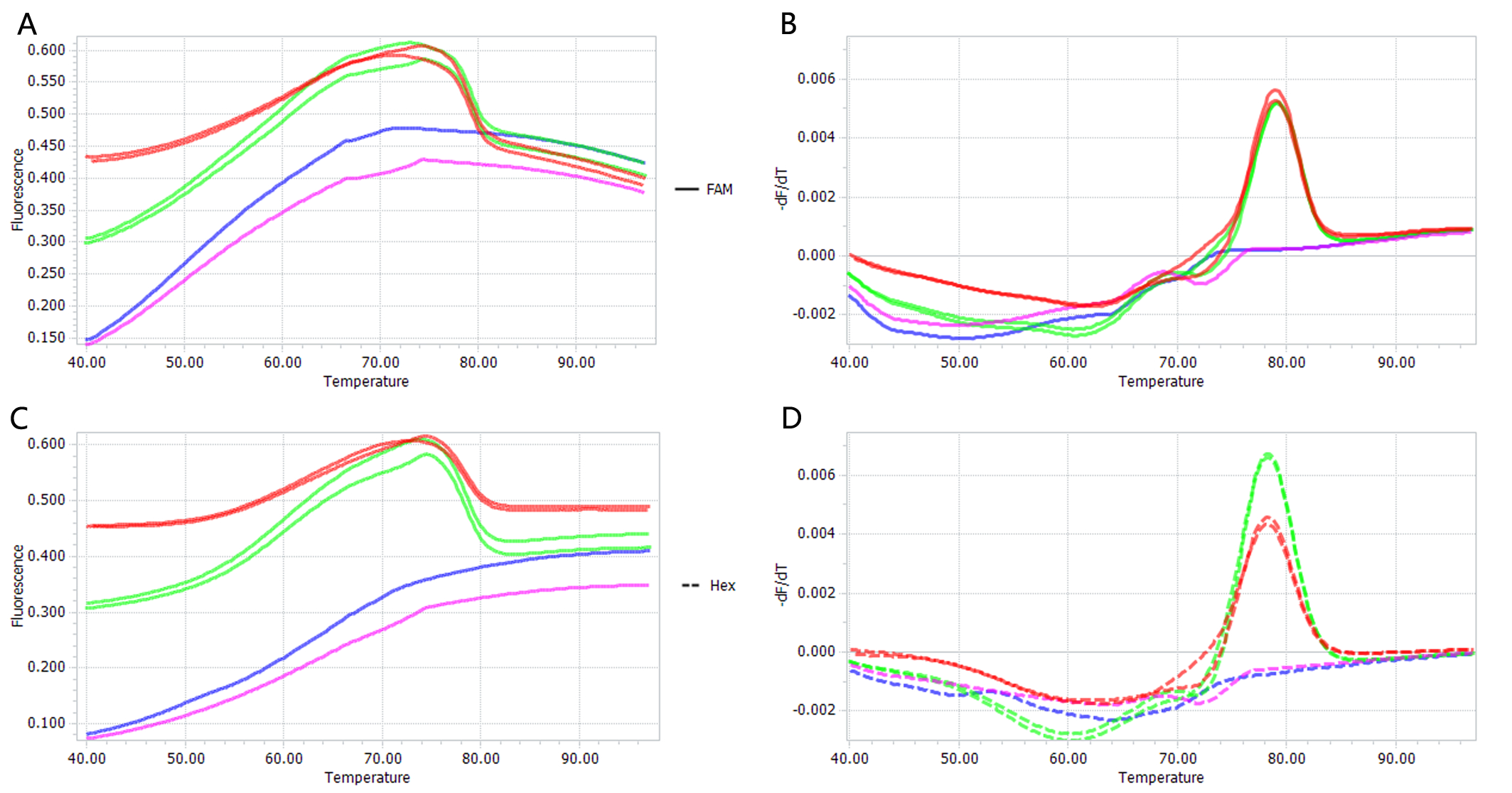

Supplement: Supplementary file 5 — Influence of probe hydrolysis on the melting curve profile. (A) Melting curves and (B) Melting peaks in the FAM channel, and (C) Melting curves and (D) Melting peaks in the HEX channel for recombinant plasmid p-H. Red and green lines indicate the addition of probes P1 and P2 before and after PCR for plasmid p-H, and blue and pink lines indicate the addition of probes P1 and P2 before and after PCR for NTC. (TIF 1001 kb) [file 12917_2018_1697_MOESM5_ESM.tif]
